# Structural analysis of FAD synthetase from *Corynebacterium ammoniagenes*

**DOI:** 10.1186/1471-2180-8-160

**Published:** 2008-09-23

**Authors:** Susana Frago, Marta Martínez-Júlvez, Ana Serrano, Milagros Medina

**Affiliations:** 1 Departamento de Bioquímica y Biología Molecular y Celular, Facultad de Ciencias and Institute of Biocomputation and Physics of Complex Systems, Universidad de Zaragoza, Zaragoza, Spain

## Abstract

**Background:**

The prokaryotic FAD synthetase family – a group of bifunctional enzymes that catalyse riboflavin phosphorylation and FMN adenylylation within a single polypeptide chain- was analysed in terms of sequence and structure.

**Results:**

Sequences of nearly 800 prokaryotic species were aligned. Those related with bifunctional FAD synthetase activities showed conservation of several consensus regions and highly conserved residues. A 3D model for the FAD synthetase from *Corynebacterium ammoniagene*s (*Ca*FADS) was generated. This model confirms that the N-terminal and C-terminal domains are related to nucleotydyltransferases and riboflavin kinases, respectively. Models for the interaction of *Ca*FADS with its substrates were also produced, allowing location of all the protein substrates in their putative binding pockets. These include two independent flavin binding sites for each *Ca*FADS activity.

**Conclusion:**

For the first time, the putative presence of a flavin binding site for the adenylylation activity, independent from that related with the phosphorylation activity, is shown. Additionally, these models suggest the functional relevance of some residues putatively involved in the catalytic processes. Their relevant roles were analysed by site-directed mutagenesis. A role was confirmed for H28, H31, S164 and T165 in the stabilisation of the P groups and the adenine moiety of ATP and, the P of FMN for the adenylylation. Similarly, T208, N210 and E268 appear critical for accommodation of the P groups of ATP and the ribityl end of RF in the active site for the phosphorylation process. Finally, the C-terminal domain was shown to catalyse the phosphorylation process on its own, but no reaction at all was observed with the individually expressed N-terminal domain.

## Background

The activities of riboflavin (RF) phosphorylation and FMN adenylylation are present among all kingdoms of living organisms. However, whereas eukaryotes use two different enzymes for FMN (flavokinase) and FAD (adenylyltransferase) production [1-6], prokaryotic organisms depend on an enzyme of ~38 kDa, FAD synthetase (Flavin adenine dinucleotide synthetase, FADS), that exhibits both activities [7]. Thus, FADS constitutes a bifunctional prokaryotic protein family of enzymes with both, ATP:RF 5'-phosphotransferase [Riboflavin kinase (RFK) (EC 2.7.1.26) (Flavokinase)] and ATP:FMN-adenylyltransferase [FMN adenylyltransferase (EC 2.7.7.2)] activities [7,8] that catalyses the 5'-phosphorylation of RF to FMN and, subsequently, the adenylylation of FMN to FAD.

RF phosphorylation      RF + ATP → FMN + ADP

FMN adenylylation      FMN + ATP → FAD + PP_i_

The FMN adenylylation reaction of FADS is a reversible process, while phosphorylation of RF appears to be irreversible [9]. Additionally, FADS has been shown to function with a broad variety of RF isoesters, and it is, therefore, widely used in the preparation of FMN and FAD analogues [8,10]. A kinetic mechanism for the steady-state reaction of the FADS from *Corynebacterium ammoniagenes *(*Ca*FADS) has been proposed [9], suggesting an order in substrate binding and product release. The FMN intermediate produced by the first reaction appears to be released by the enzyme to later rebind the enzyme as a substrate for FAD production. Both enzyme activities present important differences in their substrate requirements, including concentration dependence, specificity for divalent cations and optimal pH or temperature [7,11,12].

The C-terminal region of FADS (~150 residues) shares considerably sequence similarity with eukaryotic monofunctional RFKs. Likewise, the N-terminal region has been proposed to present remote similarity to nucleotydyltransferases (NTs), suggesting its involvement in the adenylylation process [13]. The structure of bifunctional FADS has only been reported for the *Thermotoga maritima *enzyme (*Tm*FADS), both free and in complex with some substrates [14,15]. This structure shows that the enzyme is folded in two domains and it also confirms the presence of one ATP-binding site in each of the domains and a single flavin-binding site [14,15]. So far, neither functional studies of the *Tm*FADS nor FADS structures from any other species have been reported.

A sequence analysis of the bifunctional FADS family and an *in silico *structural model for the *Ca*FADS are here presented. Based on these data, a preliminary functional analysis of the *Ca*FADS has been performed. Several residues are shown to be exclusively involved in either the first or second catalytic event. Additionally, the C- and N-terminal domains have been independently produced and assayed for their activities. The structural information here provided might help to envisage the rational design of selective antimicrobial drugs with the function of inhibiting FMN or FAD production and therefore, the availability in the cell of enzymes depending on these cofactors.

## Methods

### Sequence searches and alignments

**Figure 1:**
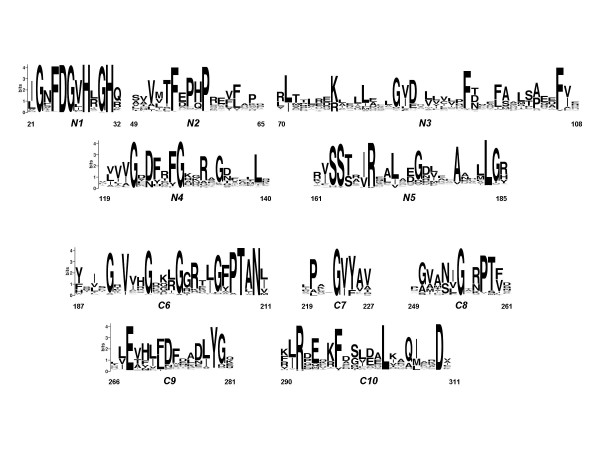
**Sequence logos including the bifunctional FADS family consensus regions and conserved residues.** Numeration of *Ca*FADS is used. The data of this logo is based on 500 sequences from the bacteria+archaea alignment of the FADS family. The sequence logo was produced using the WebLogo server [18].

The primary structure of *Ca*FADS (sp|Q59263|RIBF_CORAM) was obtained from the UniProtKB/SwissProt database [12]. A SIB-BLAST search of the complete *Ca*FADS amino acid sequence was performed at [16] against the Eukaryota and Bacteria+Archaea subsections of the non-redundant data base UniProt Knowledgebase (Swiss-Prot + TrEMBL). Multiple sequence alignment was generated using the CLUSTAL W algorithm  with default parameters [17]. Graphical representation of the multiple sequence alignment was visualised using the WebLogo server  (Figure 1) [18]. The sequence numbering used throughout the paper corresponds to *Ca*FADS.

### Prediction of a three-dimensional model for the structure of FAD synthetase from *C. ammoniagenes*

The structural model for *Ca*FADS was constructed using homology modelling procedures based on multiple structure-base sequence alignments (including all the proteins in the PDB) as implemented in the Geno3D Web server [19]. This program uses distance geometry, simulated annealing and energy minimisation algorithms to build the protein models. The structural quality of the models was checked using the PROCHECK validation program [20]. The structures of *Tm*FADS (1mrz, 1s4m, 1t6x, 1t6y) [14,15] and those of RFKs from *H. sapiens *(1q9s, 1p4m) [21,22] and *S. pombe *(1n06, 1n08) [23] were suitable as templates. Six different combinations of templates were used and three models were generated by each combination.

### Prediction of substrate binding sites

Predictions of putative ATP, Mg^2+ ^and FMN binding sites of *Ca*FADS for FMN adenylylation at the N-terminal domain were carried out by comparing our models with the structures of the nicotinamide mononucleotide adenylyltransferases (NMNAT) in complex with their substrates (Table 1). Similarly, to model ATP, Mg^2+ ^and RF binding to the C-terminal domain of *Ca*FADS, the structures of two RFKs in complex with their substrates (Table 1), as well as the different structures reported for *Tm*FADS bound to its different substrates (Table 1), were used. O [24], Spdb-Viewer 3.7 [25] and PyMol 0.99 softwares [26] were used for complex modelling, structural analysis and figures production.

**Table 1:** Structures used to model *Ca*FADS and the interaction with its ligands.

	PDB	SUBSTRATES	(%) IDENTITIES	(%) CONSERVATIVE SUBSTITUTIONS	REFERENCE
FAD synthetases					
*T. maritima*	1mrz		29.3	40.2	[14]
N-terminal^a^			30.6	39.2	
C-terminal^b^			27.6	41.5	
*T. maritima *complexed					
	1s4m	RF	29.3	40.2	[15]
	1t6x	ADP			
	1t6y	FMN, ADP, AMP			
Nucleotydyltransferases^a^					
GCT *B. subtilis*	1coz	CTP	12.4	23.1	[30]
NMNAT *M. jannaschii*	1f9a	Mg^2+^, ATP	12.9	22.0	[32]
NMNAT *H. sapiens*	1kku		10.8	18.3	[31]
NMNAT *M. thermoautotropicum*	1ej2	NAD^+^, Na^+^	13.0	22.6	[33]
PPAT *E. coli*	1b6t	dPCoA	13.0	26.3	[34]
	1gn8	ATP, Mn^2+^			[37]
PPAT *T. thermophilus*	1od6	Ppant	11.3	22.0	[35]
Riboflavinkinases^b^					
*H. sapiens*	1p4m	ADP, Mg^2+^, FMN	25.7	41.4	[21]
	1q9s	ADP, Mg^2+^, FMN			[22]
	1nb0	ADP, Mg^2+^			[21]
*S. pombe*	1n06	ADP			[23]
	1n07	FMN, ADP	23	38.2	
	1n08	Zn^2+^, ADP			

Parameters derived from the structure-based sequence alignment of the produced *Ca*FADS models over structural databases.

^a ^Alignments with the segment 19–186 from *Ca*FADS

^b ^Alignments with the segment 187–338 from *Ca*FADS

### Cloning and production of *C. ammoniagenes* wild-type and mutated FAD synthetase forms in *E. coli*

Following the Ethical Rules established at the Universidad de Zaragoza, strain DSM 20305 (ATCC 6872) from *C. ammoniagenes *was grown at 30°C in a culture medium containing 0.5% glucose, 0.5% NaCl, 1% tryptone and 0.5% yeast extract, pH 7.2–7.4. Genomic DNA was isolated from the cells as previously described [27]. The *Ca*FADS encoding sequence was then amplified by the polymerase chain reaction using the forward 5'-GTAAGCCATGGATATTTGGTACGGAACAG-3' and reverse 5'-CCATCGATAGCGGATCCGGCATATAC-3' primers, synthesised on the basis of the published nucleotide sequence of the enzyme [12]. The primers incorporate the *NcoI *and *BamHI *sites respectively, and have the transcription start codon GTG replaced with ATG. The amplified 1237 bp DNA fragment was then cloned in the *NcoI*/*BamHI *sites of the pET-28a(+) expression vector. The resulting recombinant plasmid, PET28a-FADS, was used to transform *E. coli *BL21(DE3) competent cells. The transformants were selected on solid LB/agar medium containing 30 μg/ml kanamicin. The fragment was sequenced to confirm the accuracy of the amplification. Mutations were introduced into the cloned structural gene encoding the WT *Ca*FADS by site-directed mutagenesis. The H28A, H28D, H31A, H31D, R161A, R161D, S164A, S164D, T165A, T165D, T208A, T208D, N210A, N210D, E268A and E268D FADS mutants were generated using the QuikChange mutagenesis kit (Stratagene) in combination with the adequate synthetic oligonucleotides. The gene codifying for the individual N-terminal domain (PET28a-Δ(183–338)FADS) was obtained replacing the codon codifying for Leu183 in PET28a-FADS with a stop codon, following the methodology above mentioned for production of site-directed mutants. The C-terminal domain was individually cloned (PET28a-Δ(1–182)FADS) using the protocol described for WT *Ca*FADS with the forward primer 5'-CCAACTGGGCCATGGGGCGGCAC-3' and the reverse primer above used for the full length protein cloning. The forward primer used to clone the C-terminal domain incorporates the start codon ATG in a *NcoI *site substituting for Leu183. Mutations were verified by DNA sequence analysis. WT and mutant FADS proteins were over-expressed in *E. coli*.

### Purification of recombinant FADS from *C. ammoniagenes*

*E. coli *cells containing the recombinant pET28a-FADS plasmid (or its mutants) were grown in LB. During the exponential *E. coli *growing phase, FADS was induced by overnight incubation at 37°C with IPTG (1 mM). Cells were harvested by centrifugation and stored at -20°C. In a typical purification, around 25 g of cells (over-expressing recombinant FADS) were thawed, resuspended in 125 ml of cell disruption buffer (50 mM Tris/HCl, pH 8.0, 1 mM EDTA, 12 mM *β*-mercaptoethanol, 1 μM PMSF) and broken by ultrasonic treatment at 4°C using 16 cycles of 1 min in a DRH UP200 DR Hielsher sonicator. The cell debris was removed by centrifugation at 40000 g during 45 min. The resulting yellow FADS-containing supernatant was fractioned with 45% ammonium sulphate. The mixture was again centrifuged for 45 min at 4000 g and the supernatant was loaded onto a Phenyl-Sepharose High performance (Amersham Biosciences, GE Healthcare) column equilibrated with 50 mM Tris/HCl, pH 8.0, 45% ammonium sulphate. The column was washed with the same buffer containing only 17% ammonium sulphate until most of the yellow colour washed out of the column. The enzyme was eluted using a 17→0% ammonium sulphate reversed-gradient in the same buffer. Fractions of 6–8 ml were collected while the absorbance at 280 nm was recorded. Fractions presenting absorbance at 280 nm were analysed by SDS-PAGE and, those containing the over-expressed FADS were pooled and dialysed against 50 mM Tris/HCl, pH 8.0. After dialysis the protein was loaded onto a DEAE-cellulose (DE52, Whatman, England) column equilibrated with 50 mM Tris/HCl, pH 8.0. The column was washed with the equilibration buffer containing 0.1 M NaCl and subsequently eluted using a 0.1→0.5 M NaCl gradient. Fractions of 5 ml were collected while recording the absorbance at 280 nm and, subsequently analysed by SDS-PAGE. The enzyme was considered pure when a single band of the expected size was observed in SDS-PAGE. Pure FADS fractions were pooled together, dialysed against 50 mM Tris/HCl, pH 8.0 and stored at -20°C.

### Measurement of riboflavin kinase and adenylyltransferase activities of FAD synthetase

Conversion of RF into FMN and, of FMN into FAD were qualitatively assayed by addition of the different FADS variants (final enzyme concentration ~1 μM, calculated using a theoretical ε_280 nm _= 27.8 mM^-1^cm^-1^) to a solution (final volume, 150 μl) containing 50 μM flavin (either RF or FMN), 0.2 mM ATP and either 0.8 mM or 10 mM MgCl_2_, in 50 mM Tris/HCl, pH 8.0. All these chemicals were obtained from SIGMA and were from the highest purity available. After 30 minutes of incubation at 37°C, the reaction was stopped by boiling the preparations for 5 minutes. Transformation of RF into FMN and FAD, or FMN into FAD, was visualised by resolving the products of the reaction at room temperature and in the dark by TLC on Silica Gel SIL-G-25 (20 cm × 20 cm, thickness 0.25 mm) plates [28]. The moving phase was a solution of butanol:acetic acid:water (12:3:5). Flavin TLC spots were visually examined and scanned by determining their fluorescence under an ultraviolet light. The different flavins move up the plate at different rates due to differences in their partioning behaviour between the mobile liquid phase and the stationary phase. The weaker intensity of the FAD fluorescence with regard to equivalent amounts of RF or FMN relates with its flavin fluorescence quenching upon stacking of the adenine portion [29]. An additional band, running slightly faster than FMN, is observed in some experiments. The nature of this band is not known, but it is only present in the experiments containing simultaneously FMN and FAD.

## Results and discussion

### Sequence analysis of the FAD synthetase family

No matches for bifunctional FADS sequences were obtained when performing the SIB-BLAST search of the *Ca*FADS sequence on the UniProt Knowledgebase eukaryota subsection. When searching the bacteria+archaea database subsection (2,929,087 sequences searched), the 777 sequences displaying E-values ≤ 0.009 were chosen for further analysis. Redundant sequences from the same species presenting sequence identity over 80% were eliminated. The remaining sequences included some proteins noticeably shorter as well as proteins lacking the typical consensus sequences found in FADSs. These sequences (less than 5%), most of them displaying the lowest E-values of the selected sequences, were removed and separately analysed. They could be sorted out into several groups: i) proteins that present only the N-terminal region of FADS, ii) proteins that present only the C-terminal region of FADS, iii) proteins that lack the first conserved motif in the N-terminal region, iv) proteins with a conserved N-terminal region and a C-terminal region that shares no similarity with the bifunctional FADS family, and v) proteins with a conserved C-terminal region and a N-terminal region that shares no similarity with the bifunctional FADS family (in *Rhodococcus sp*. RHA1, the protein N-terminal region is 40% identical to 3,4-dihydroxy-2-butanone-4-phosphate synthase, an enzyme involved in RF biosynthesis). Some of these divergent proteins belong to organisms that also possess a typical prokaryotic FADS sequence.

After the manual refinement, a multiple sequence alignment over the remaining 500 FADS-like sequences, displaying E-values ≤ 2xe^-6 ^with *Ca*FADS, was carried out. Only FADSs from the genus *Corynebacterium *displayed sequence identities over 50% with the *Ca*FADS (up to 60% identity in the case of the FADS from *C. efficiens*). However, the multiple alignment pointed out several highly conserved residues and motifs (Figure 1). Motifs 21-Φ-G-Ω-F-D-G-Φ-H-Ω-G-H-Ω-32 and 162-Φ-S-S-[TS]-Ω-[IV]-R-Ω-Ω-Φ-Ω-Ψ-G-174 (Φ, Ψ and Ω, denoting hydrophobic, polar and any residue, respectively) (N1 and N5 in Figure 1) corresponded to two consensus sequences in the FADS family that are also conserved in NTs remotely related to FADS (see below). Two additional consensus sequences in between the above mentioned motifs were also observed. They included residues 50-Ω-Φ-Φ-Ω-F-Ψ-P-[HQ]-P-Ω-59 and 120-Φ-Φ-Φ-G-Ω-[DN]-[FYH]-Ω-[FY]-G-Ω-129 (N2 and N4 in Figure 1) and, apparently, only the motif 123-G-Ω-Ψ-125 presents an equivalent in NT sequences (see below). Multiple sequence alignment also showed a number of residues highly conserved, or conservatively substituted, between residues 1 and 186: I71, R77, G85, I86, D87, F94, F98, Y106, V107, A179, L183 and G184 (*Ca*FADS numbering). These residues are mainly included in region N3 and at the end of N5 in Figure 1 and, can be considered as forming additional consensus sequences. Noticeably, only R77 and A179 are conserved in NTs.

The C-terminal region of the bifunctional FADS sequences (residues 187–338) also included several consensus sequences, 191-G-Ω-V-Φ-Ψ-G-Ω-Ω-Ω-G-Ω-Ψ-Φ-203, 205-G-[FY]-P-T-[ALIV]-N-210, 223-P-Ω-Ω-G-[VI]-[YF]-225, 253-Ψ-Φ-G-Ω-Ψ-P-T-[FVI]-260, 267-[LVI]-E-Ω-[HFYN]-Φ-[FL]-[DN]-[FWY]-Ψ-Φ-[DNE]-[LIAV]-Y-[GDN]-280 and 291-[LI]-R-Ω-[EQMN]-Ω-[KRT]-[FY]-296, and the highly conserved residues L303 and D310 (see C6–C10 in Figure 1), being all of them also conserved in the sequences of RFKs (see below).

### Structural model of the FAD synthetase from *C. ammoniagenes*

**Figure 2:**
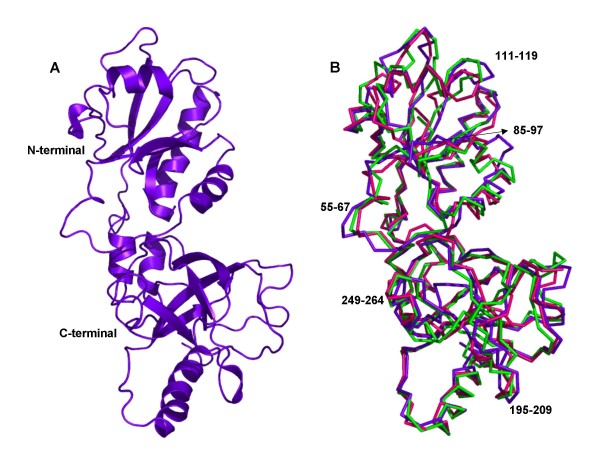
**Predicted structural model for *Ca*FADS. **(A) Ribbon representations of the model selected to represent *Ca*FADS (Table 2). (B) Superposition of the three models generated in this prediction. Residues involved in putatively high flexible regions are indicated.

**Figure 3:**
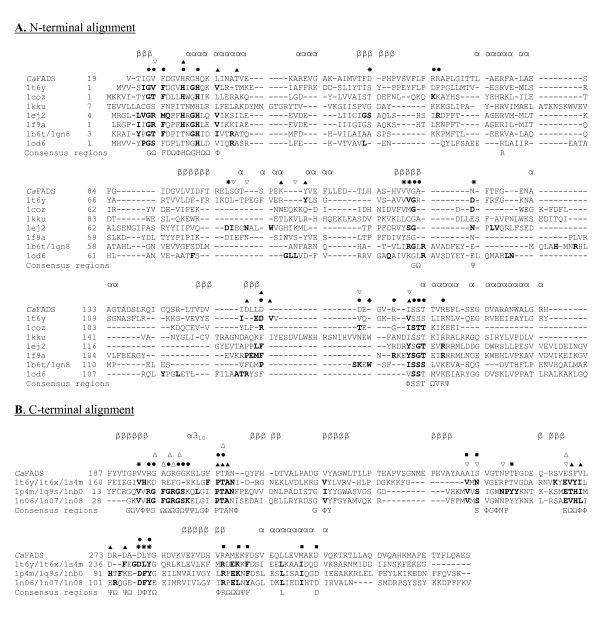
**Alignments. **(A) Structural alignment of the N-terminal domain of the *Ca*FADS model (residues 19–186) with the corresponding regions of *Tm*FADS and several NTs. Residues proposed to be involved in particular substrate binding are marked as; ● for phosphate groups, ✳ ribose or ▲ adenine portions of ATP. ▽ denotes regions where substrates specific for each enzyme have been reported to interact. (B) Structural alignment of the C-terminal domain (residues 187–338) of the *C. ammoniagenes *FADS model with those of *Tm*FADS and RFKs from *S. pombe *and *H. sapiens*. Residues proposed to be involved in particular substrate binding are marked as; ● for phosphate groups, ✳ ribose or ▲ adenine portions of ATP and, △ for the phosphate, ▽ ribityl or ■ isoalloxazine ring portions of RF. Each template is referred to its pdb code according to Table 1. Residues involved in substrate binding in each particular structure are shown in bold case. Consensus sequences are shown below alignment, Φ, Ψ and Ω denoting hydrophobic, polar and any residue, respectively. Secondary structure prediction of *Ca*FADS, obtained using the JOY server [36] and visual inspection, is shown above each alignment, with β signifying β-strand residues and α signifying α-helix residues.

A 3D structural model for the *Ca*FADS has been produced based on the structure of the *Tm*FADS (Figure 2) [14,15]. The low sequence similarity of the N-terminal domain of FADSs with NTs prevented these enzymes to be used as templates (Table 1, Figure 3A) [13], but the sequence similarity found at the C-terminal domain of the FADS family with RFKs from *H. sapiens *[21,22] and *S. pombe *(Table 1, Figure 3B) [23] allowed them to be used as templates. These RFKs were used for the construction of the model in those loops were the X-ray structure of *Tm*FADS did not show experimental information [14,15]. Models for the *Ca*FADS C-terminal domain generated using as templates only the structures of RFKs were consistent with the C-terminal structure reported for *Tm*FADS, indicating that RFKs are a good choice as templates for this FADS family domain. Stereochemistry and energy analysis of the produced models suggested that the model constructed using the FADS and RFK templates reported in Table 2 (Figure 2A) was adequate to describe the 3D structure of *Ca*FADS until structures are provided by experimental methods.

**Table 2:** R.m.s.d. (Å) between the three predicted models for *Ca*FADS and the structures used for their production.

Structure	Model 1	Model 2	Model 3
Model 1	0.00	1.34	1.49
Model 2	1.34	0.00	1.22
Model 3	1.49	1.22	0.00
*T. maritima *FADS (1mrz)	1.53	1.49	1.44
*T. maritima *FADS, RF (1s4m)	1.60	1.56	1.39
*H. sapiens *RFK, ADP, Mg^2+^, FMN (1p4m)	1.74	1.54	1.25
*S. pombe *RFK, ADP, Zn^2+ ^(1n08)	1.82	1.67	1.60

According to the model, the protein folds in two domains. The N-terminal domain (residues 18–186) lacks the first 18 residues of the sequence (not present in the *Tm*FADS template), while the C-terminal domain (residues 187–318) lacks the last 20 residues (templates were shorter in sequence or presented very low sequence similarity in those residues) (Figures 2 and 3). The main structural arrangements predicted for the *Ca*FADS are present in the structure reported for the *Tm*FADS [14,15], but our model also showed the disposition of some loops and a 3_10_-helix at the C-terminal domain that are missed in the *Tm*FADS structure. Additionally, the C-terminal domain of *Ca*FADS showed an insertion around residue 230 with regard to *Tm*FADS and many members of the FADS family.

### The kinase domain: the C-terminal domain of *C. ammoniagenes* FAD synthetase

**Figure 4:**
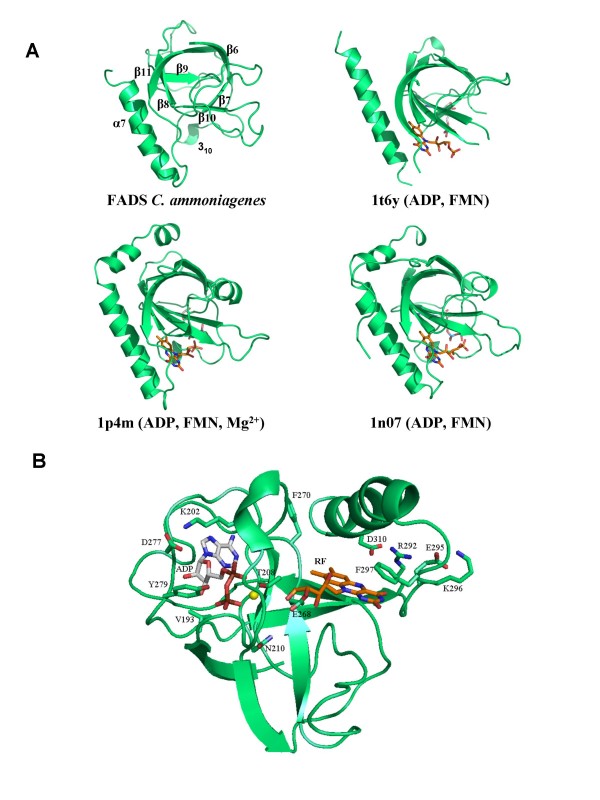
**C-terminal domain structure of*****Ca*****FADS.** (A) Comparison of the structural elements with those found in RFKs. (B) Model for the interaction of ADP-Mg^2+ ^and RF with the C-terminal domain. Residues putatively involved at the RFK active site, nucleotides and flavins are shown in CPK sticks. Carbon atoms are shown in green, white and orange for protein side-chains, nucleotides and RF, respectively. Mg^2+ ^ions are shown as yellow balls.

The C-terminal domain of the *Ca*FADS model (residues 187–318) folds in a central six-stranded antiparallel β-barrel stabilised between the single final α-helix (α7), the two small α5 and α6 of the N-terminal domain and several loops (Figure 2A). A short 3_10_-helix is also predicted in our model between residues G201-L204. This C-terminal domain showed a high sequence identity in the 3D space with the structures of the templates (Figures 3B and 4A, Table 1) and, there is no doubt that it must contribute to ATP, Mg^2+ ^and RF binding to produce FMN. Comparison of this model with the reported *Tm*FADS and RFKs structures in complex with their substrates will provide information about *Ca*FADS residues involved in substrate binding and catalysis (Figures 3B and 4B). Therefore, it is expected that the ADP-Mg^2+^, or ATP-Mg^2+^, will be nested at one edge of the β-barrel, between two loops (Figure 4B). The first loop (R195-N210) contains the short 3_10_-helix and the 207-PTΦN-210 consensus sequence conserved in RFKs (Figures 1 and 3B) and, it is proposed to stabilise the P groups (Figure 4B). The structures of RFKs in complex with ADP-Mg^2+ ^also suggest that T208 and N210 might contribute to metal binding [22,23]. The second loop (E268-Y279) starts with the E268 residue, invariant in FADS and RFK families and proposed to link the terminal OH group of the RF ribityl chain, acting as a catalytic base [21-23]. This loop also includes the D277 and Y279 conserved positions, proposed to stabilise the ATP adenine ring, while V193, K202 and D277 might contribute to stabilise the ribose (Figures 1 and 4B) by similarity with RFKs [21,23]. Comparison of models with *Tm*FADS and RFKs complexed with RF or FMN suggests that the isoalloxazine ring must be bound in a pocket formed by the outer surface of the β-barrel (β8–β10) and the long terminal α7 helix. The flavin ring might result stabilised by the hydrophobic environment provided by the consensus region 223-G-V-Y-225 and the conserved F206, F270, F297, L303 and M307 residues, while its hydrophilic portion may be pointing towards the opening of the pocket and stabilised by E295 or K296 [15,21,22]. Other residues apparently not directly involved in flavin binding but contributing to the formation of the bottom and top of the pocket include the consensus region 254-G-Ω-Ψ-P-T-[FVI]-260 and the highly conserved residues R292 and D310, respectively. These two later residues apparently form a salt bridge that contributes to fix the terminal α-helix close to the top of the flavin ring cavity (Figure 4B). The ribityl chain would extend towards β7, interacting as above mentioned with E268 (Figure 4B).

The models for *Ca*FADS suggest flexibility in the loops connecting β6–β7 and β9–β10 (Figure 2B). Both of these regions are not solved in the structures reported for the *Tm*FADS [14,15]. Flexibility is also observed in regions involved in substrate binding (Figure 4B), in agreement with conformational changes observed in RFKs upon substrate interaction [22]. These conformational changes provide additional interactions, for both the ribityl tail and the isoalloxazine ring and, help to anchor the reactive substrates and the catalytic residues in the most adequate disposition for catalysis [21-23]. This analysis also suggests that despite RFKs and FADSs surely share a similar mechanism in the RF phosphorylation reaction, variations in the structural details for substrate binding and catalysis can be expected.

### The adenylyltransferase domain: the N-terminal domain of *C. ammoniagenes* FAD synthetase

**Figure 5:**
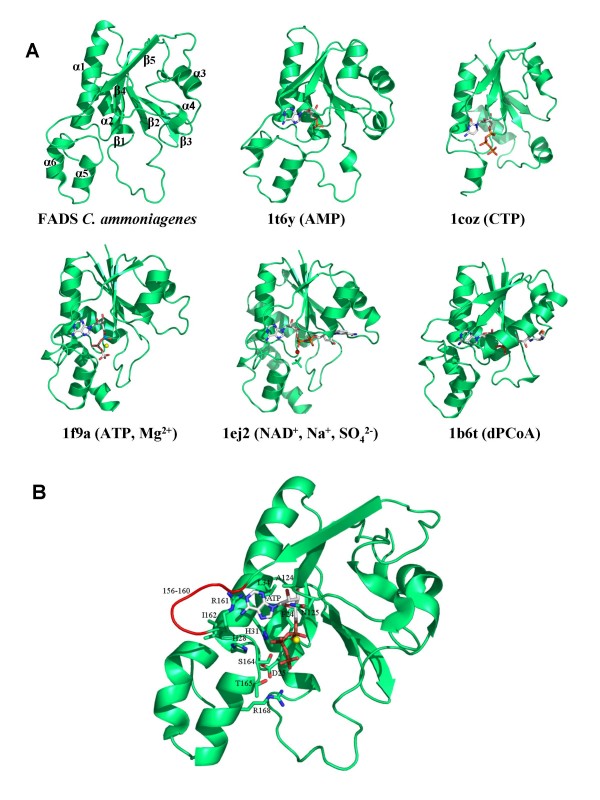
**N-terminal domain structure of *Ca*FADS.** (A) Comparison of the structural elements with those found in NTs. (B) Model for the interaction of ATP-Mg^2+ ^with the N-terminal domain. Residues putatively involved in the NT activity and ATP binding are drawn as sticks and CPK coloured. Carbon atoms are shown in green and white, for the protein side-chains and nucleotides, respectively. Mg^2+ ^and Na^+ ^ions are shown as yellow and red balls, respectively.

**Figure 6:**
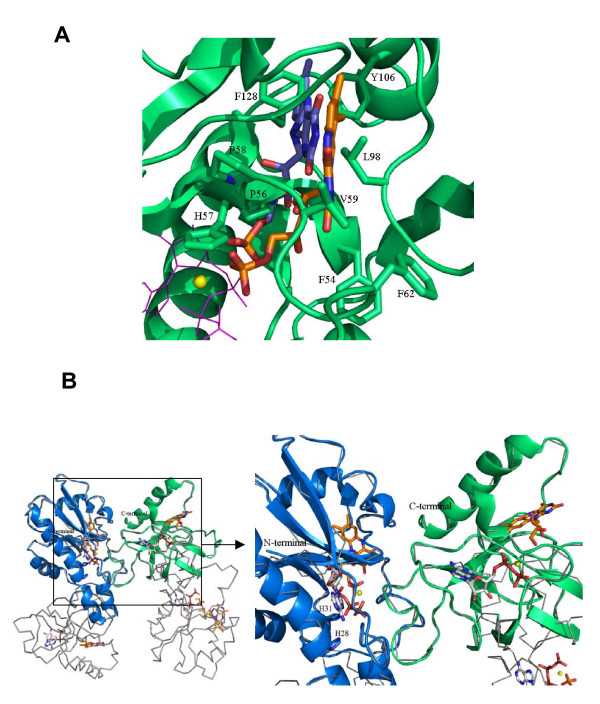
**Localisation of the flavin binding site at the N-terminal domain of CaFADS. **(A) Models for the interaction of FMN with the N-terminal domain. FMN and residues putatively involved in its stabilisation are drawn in sticks and CPK coloured. Two possible conformations for the FMN are indicated (Carbon atoms are in orange and blue, respectively). Positions of ATP and Mg^2+ ^are also indicated as pink lines and a yellow ball, respectively. (B) Model for the interaction between two *Ca*FADS monomers (grey lines), based on the *Tm*FADS crystallographic dimeric crystal unit. The C-terminal of monomer 1 is shown in green and the N-terminal of monomer 2 in blue. Position of the RF and FMN substrates, as well as ATP-Mg^2+^, are indicated at each binding site in CPK. Carbon atoms are shown in white and orange for nucleotides and flavins, respectively. H28 and H31 are shown as sticks.

The *Ca*FADS N-terminal domain consists of a classical α/β dinucleotide binding domain. Its core is formed by a twisted five-stranded parallel β-sheet flanked by two long and two short α-helices and the corresponding connecting loops (Figure 5). The end of this domain forms a small subdomain made up of two small α-helices (α5 and α6), which are in contact with the C-terminal domain. The *Ca*FADS model was compared with the structures reported for several enzymes of the NT α/β phosphodiesterase superfamily (Table 1, Figures 3A and 5A), either free or in complex with their substrates [13-15,30-35]. Members of this family catalyse the transfer of a nucleotide monophosphate moiety, from a nucleotide triphosphate (NTP), onto different substrates. Our *Ca*FADS N-terminal model presents an overall folding conserved in NTs (Figure 5A) that allowed improvement of the sequence alignments and production of putative models for the interaction between this *Ca*FADS domain and its substrates (Figures 3A, 5 and 6). The consensus regions 19–32 and 162–174, highly conserved in the FADS and NT families (Figures 1 and 3A), are proposed to be involved in NTP stabilisation in the active site. In our model these motifs include the α1 helix (H28-V45), placed between β1 and β5, the E158-S164 loop and some portions of the two terminal antiparallel α-helices, (α5 (T165-S172) and α6 (D175-L183)) (Figure 5). Residues of these consensus regions show a high conservation in primary and 3D structures when comparing the FADS and NT families. Thus, in *Ca*FADS: i) main-chain atoms of G22, F24 and D25 might H-bond the α-P of ATP, ii) H28, G30 and H31 (N-terminal of helix α1) might contribute to stabilise the P groups and adenine moiety of ATP, iii) the ATP P groups might interact with L34, T38, I162, G174 and the 104–106 and 153–157 regions, iv) the consensus region 162–165 (including the N-terminals of α5) must be involved in stabilisation of the adenine, β-P and γ-P of ATP and, v) the 123–125 consensus motif accommodates the ribose portion of the nucleotide (Figures 3A and 5), as proposed for the equivalent residues in *Tm*FADS, glycerol-3 phosphate cytidylyltransferase (GCT), phosphopantetheine adenylyltransferase (PPAT) and NMNAT (Figure 3A, 5A) [31,32,34]. The loop formed by residues 156–160 (in red in Figure 5B), although conserved in sequence and structure, shows different orientations and lengths in the different NTs, which probably determines the enzyme specificity for the NTP (Figures 3A and 5A) [30]. The *Ca*FADS model also showed several positively charged side-chains highly conserved in the FADS family and structurally situated in positions also occupied by positively charged side-chains in NT structures. These include R161 and R168 (R161 provided by R91 and R22 in PPAT from *E. coli *or *T. thermophilus *and, R168 by K46 in *B. subtilis GCT *or R47 in *M. thermoautotrophicum *NMNAT). Their positions suggest that these side-chains might contribute to stabilise the adenine and P groups of ATP when bound to the active site. It is also worth noting that an important number positions occupied by conserved glycines in the FADS family are also conserved in sequence and structure in NTs and, in many cases, their main-chain atoms directly provide H-bonding to the nucleotide (Figures 1 and 3A).

Flexible regions can be predicted by comparison of the *Ca*FADS models (Figure 2B, Table 2). These, including 55–67, 85–97, 111–119 and 127–135, correspond to low similarity regions between NT and FADS families (Figures 2B, 3A and 5A). Structures of NMNAT in complex with NAD^+ ^[33], and PPAT in complex with dPCoA [34] or Ppant [35], indicate that the binding of the adenylylation substrate, NMN^+^ or Ppant moieties, is allocated in the pocket formed by the above mentioned regions. Superposition of these complexes on our *Ca*FADS model, followed by fitting and sculpting the FMN molecule along the nicotinamide nucleotide moiety of NAD^+ ^in the NMNAT structure, allowed production of putative models for the interaction of the FADS N-terminal domain with its FMN substrate (Figure 6A). In this model, the FMN isoalloxazine ring would be placed in a cavity surrounded by the above mentioned flexible regions. The entrance of this cavity would be flanked by the F54, F62 and D94 hydrophobic side-chains, the consensus region 54-FPHP-58 would fix the position of the ribityl of FMN, while the flavin ring environment would be contributed by hydrophobic interactions with V59, L98, Y106 and F128 (Figure 6A). All these positions are highly conserved in the FADS family, but do not have equivalence in NTs (Figures 1 and 3A). The model suggests that the hydrophobic isoalloxazine portion of the flavin ring might be embedded inside the cavity, while the hydrophilic edge would be partially exposed to the solvent. The size of the cavity makes it difficult to predict the precise location of the isoalloxazine ring. Various possibilities are allowed (Figure 6A) and, final disposition of loops and side-chains would depend on the flavin binding event and conformational changes not predicted in our model. This flavin binding mode could represent a novel flavin binding motif so far not described. Additionally, while sequence motifs N1, N4 and N5 are also conserved in the sequences of eukaryotic FMN adenylyltransferases, residues and regions proposed for flavin binding in the FADS family do not present homologous in the sequences of the monofunctional enzymes (data not shown).

This is the first structural model where a putative binding site for the flavin is suggested at the adenylylation site of FADS, indicating the presence of two independent flavin binding sites in the FADS family. An early hypothetical model for the active site of FADS proposed a single flavin binding site for both activities and two independent ATP binding sites [9]. No structural information of this family of enzymes was reported at that time, but that model accounted for the kinetic mechanism experimentally derived [9]. The 3D structures and models today available for FADS indicate that the phosphorylation and adenylylation sites are far away (Figure 6B). This fact is in agreement with experimental observations indicating that the FMN produced in the phosphorylation process was released from the protein, to subsequently rebind to initiate the adenylylation process [9]. Superposition of our *Ca*FADS model in complex with all its substrates with the dimer reported in the *Tm*FADS crystallographic asymmetric unit also indicates that phosphorylation and adenylylation active sites from different monomers are far away to share the FMN molecule. However, this dimeric model suggests that the 229–243 loop and the terminal α7 of the C-terminal domain might contribute to the formation of the ATP and the flavin binding pockets in the adenylylation domain (Figure 6B).

### Production of *C. ammoniagenes* FAD synthetase forms

*E. coli *BL21(DE3) cells transformed with the recombinant PET28a-FADS vector produced a high level expression of active *Ca*FADS. This *Ca*FADS was purified in complex with one of the products of its reaction, FAD, as confirmed by HPLC (data not shown). To remove the FAD a hydrophobic chromatography was required. The sequence and structural analysis suggested the presence of several residues and motifs in *Ca*FADS highly conserved in this family of enzymes and potentially involved in the *Ca*talytic sites of the RFK and NT activities. Some of these residues were mutated, namely H28, H31, R161, S164, T165, T208, N210 and E268 to Ala and Asp. These *Ca*FADS variants were expressed in *E. coli*, with yields similar to the WT FADS, and were purified to homogeneity following the same protocol. Additionally, plasmids individually expressing the N-terminal and C-terminal *Ca*FADS domains were also produced, PET28a-Δ(183–338)FADS and PET28a-Δ(1–182)FADS, respectively, and transformed in *E. coli*, showing a level of expression similar to the full length protein.

### *C. ammoniagenes* FAD synthetase residues involved in FMN and FAD production

**Figure 7:**
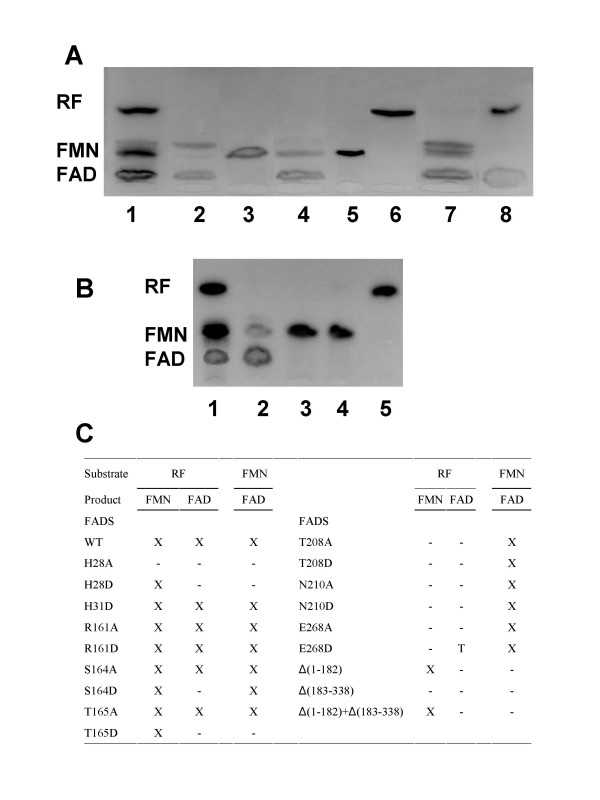
**Resolution by thin layer chromatography of the products of the RFK and the NT activities of selected *Ca*FADS variants.** The reaction mixtures contained: (A), 2-WT and 50 μM RF, 3-H28D and 50 μM RF, 4-R161D and 50 μM RF, 5-S164D and 50 μM RF, 6-T208A and 50 μM RF, 7-T208A and 50 μM FMN and 8-E268D and 50 μM RF. (B), 2-WT *Ca*FADS and 50 μM RF, 3-*Ca*FADS C-terminal domain and 50 μM RF, 4-*Ca*FADS C-terminal domain and 50 μM FMN and 5-a reaction mixture containing RF incubated in the absence of enzyme. Lanes (1) in (A) and (B) corresponded to a standard solution containing 50 μM RF, 50 μM FMN and 50 μM FAD. All the reaction mixtures contained 0.2 mM ATP and 10 mM MgCl_2 _in 50 mM Tris/HCl, pH 8.0. Experiments in (A) were carried out with pure *Ca*FADS variants at concentration 1 μM, while reaction mixtures in (B) contained crude extracts with the over-expressed *Ca*FADS C-terminal domain. (C) Product formation in the assayed samples. X, T and hyphen indicate production, detection of only traces and no production of the corresponding reaction product, respectively.

The ability of the different individual *Ca*FADS mutants to catalyse conversion of RF into FMN and of FMN into FAD was analysed by incubating the same amount of each mutant with the substrates of both enzyme activities. Reactions were carried out under two different Mg^2+ ^concentrations; one favouring the RFK activity (0.8 mM) and the other favouring the NT activity (10 mM) [7,11,12]. However, both concentrations produced similar qualitative results under the assayed conditions. The products of the reactions were analysed by TLC (Figure 7).

Replacement of H28 with either Ala or Asp and H31 with Ala prevented conversion of FMN into FAD, confirming their implication in the *Ca*FADS NT activity. Both of these His residues belong to a consensus region 27-Φ-H-Ω-G-H-Ω-32 found both in FADS and NT families (Figure 1 and 3A). Therefore, H28 and H31 will surely be major determinants in the stabilisation of the P groups and the adenine moiety of ATP in the reaction transition state in *Ca*FADS, in agreement with previous observations in NTs [31,32,34]. Replacement of H28 with Ala also prevented the conversion of RF into FMN, pointing to a possible influence of the N-terminal domain in the RFK activity. His28 appears situated at the N-terminal of α1, in contact with α5 and α6, which, according to our model, will interact with the C-terminal domain from the same polypeptide chain, but also might influence the C-terminal of the second monomer in a putative dimeric structure (Figure 6B). However, it is difficult to envisage how the H28A mutation might affect the phosphorylation reaction with the available information. This will need further structural and biochemical characterisation. Replacement of H31 with Asp produced an enzyme still able to produce FMN and FAD, suggesting that an Asp at this position somewhat replaces the function of the His. Replacements were also introduced in the second major consensus sequence shared by the FADS and the NT families, 162-ΦSST-165. Replacement of S164 or T165 with Ala produced enzyme forms able to transform RF into FMN and FMN into FAD, while the introduction of a Asp residue at such positions clearly prevented FAD formation. This latter observation was consistent with these residues being involved in the stabilisation at the NT catalytic site of the negatively charged β-P and γ-P of ATP or the ribityl end, as suggested by the above structural analysis (Figures 3A and 5B). Finally, the putative role of the structural disposition of the positive charge of R161 in the stabilisation of the adenine and α-P and β-P of ATP in the active site was studied by replacement of R161 with either Ala or Asp. The produced FADS mutants were still able to produce FMN and FAD under the assayed conditions, suggesting the positive charge at position of R161 is not playing a critical role in *Ca*FADS catalysis.

Taking into account the fact that T208, N210, and E268 are conserved in sequence and structural position in the close environment of the ATP, not only in the FADS family but also in RFKs, and that the terminal oxygen of the ribityl of RF binding sites (Figures 1, 3B and 4), a catalytic role for them in the RF phosphorylation process can be envisaged. These *Ca*FADS residues were replaced with Ala or Asp to prove such a role. All these mutations prevented FMN production, but allowed transformation of FMN into FAD, confirming their implication in the RFK activity of FADS. These data are, therefore, in agreement with the proposed structural-based role for T208 and N210 in accommodating the active portion of the ATP molecule, the P groups, and the metal in the catalytic centre (Figure 4B). The lack of FMN and FAD production when replacing E268 with Ala confirms it is a key residue in the *Ca*FADS RFK activity. Analysis of the interaction of the *Ca*FADS model with FMN (or RF) also indicated that in *Ca*FADS E268 side-chain might H-bond to the terminal OH of the ribityl chain of RF (Figure 4B). This Glu is invariant in the FADSs and RFKs families (Figures 1 and 3B). In this later family this residue has been proposed to act as a catalytic base [21-23]. Moreover, the fact that incubation of E268D with RF did not produce any accumulation of FMN, while traces of FAD were detected, is consistent with the RFK activity taking place at a very slow rate in this mutant, thus avoiding FMN accumulation. This suggests that although the introduced mutation is conservative and the Asp side-chain might still act as a base, its shorter side-chain prevents the optimal organisation of the transition state in the active centre and, therefore, reduces the catalytic efficiency of the RFK activity. Again, this is consistent with this residue not presenting substitutions, even conservative ones, in the analysed proteins. Nevertheless, other explanations might be also possible, as higher catalytic efficiency of NT activity than that of RFK or sensitivity of the TLC analysis. Further work should be done by the analysis of the kinetic parameters for both activities, as well as of the interaction parameters for the different substrates, for all these *Ca*FADS mutants to provide additional insights in their specific role in the enzyme catalytic mechanisms.

### Activity of the independently produced *C. ammoniagenes* FAD synthetase N- and C- terminal domains

Crude extracts of the *E. coli *cells expressing the individually cloned N-terminal and C-terminal domains of *Ca*FADS, Δ(183–338)FADS and Δ(1–182)FADS, respectively, were prepared and assayed for NT and RFK activities (Figures 7B, 7C). Analysis of the products of the reaction by TLC concluded that the C-terminal domain, Δ(1–182)FADS, was able to qualitatively catalyse the phosphorylation process. However, crude extracts over-expressing the N-terminal domain, Δ(183–338)FADS, were not able to catalyse the conversion of FMN into FAD. Extracts containing both domains were mixed and assayed for both reactions. Conversion of RF into FMN was produced in these samples, but they were not able to mimic *Ca*FADS in the adenylylation process. This is in agreement with a possible C-terminal domain role in contributing to the stabilisation of the flavin and ATP substrates in the corresponding N-terminal domain pockets (Figure 6B).

## Conclusion

A sequence analysis has been carried out in the bifunctional FADS family. An important number of consensus regions and conserved residues have been determined, despite the low sequence identity among members of this family. Sequence divergences found among pathogenic organisms, such as *Mycobacterium *or *Corynebacterium*, and monofunctional eukaryotic enzymes will probably be reflected in structural divergences that might affect the catalytic mechanism, making possible the design of specific antimicrobial drugs. Models have been proposed, not only for the *Ca*FADS structure, but also for the complexes formed with all its substrates. This includes de interaction with the FMN substrate at the adenylylation site, in a region not previously reported as involved in the enzyme activity and, in a pocket that might constitute a novel flavin binding motif. As expected, these models supported that the N-terminal presents similar folding to NTs and is involved in the adenylylation of FMN, whereas the C-terminal is similar to RFKs and responsible for the phosphorylation. Models of *Ca*FADS allowed identification of several residues putatively involved in the catalysis. Site-directed mutagenesis on some of these residues confirmed their participation in substrate stabilisation and/or the catalytic mechanism. The particular behaviour of some of these mutants and of the independently expressed NT and RFK domains suggests one of the domains might influence the activity in the other one. This study sheds important and new information on the structural mechanism of substrate recognition and catalysis while waiting for the experimental structure of the *Ca*FADS and provides a platform for future investigations into the mechanism of enzymes of the bifunctional FADS family.

## Authors' contributions

SF carried out the sequence alignment, contributed to production and analysis of the properties of WT FADS and some its mutants and drafted the manuscript. MM-J carried out the production of the three-dimensional FADS model structures free and in complex with ligands. AS participated in the production of the different FADS variants and in its characterisation. MM conceived of the study, and participated in its design and coordination, in the modelling of the three-dimensional structures and drafted the manuscript. All authors read and approved the final manuscript.
